# Laryngotracheitis and Coronavirus Disease 2019 (COVID-19) in a Three-Year-Old Child: A Case Report and Literature Review

**DOI:** 10.7759/cureus.60320

**Published:** 2024-05-15

**Authors:** Mahmoud A Ali

**Affiliations:** 1 Neonatology, West Virginia University, Morgantown, USA

**Keywords:** shortness of breath, epinephrine, sars-cov-2, covid-19, stridor, croup, laryngotracheitis

## Abstract

This case report details an atypical etiology of laryngotracheitis (croup) in a three-year-old child diagnosed with coronavirus disease 2019 (COVID-19). Unlike typical croup cases, the patient required hospitalization and multiple administrations of racemic epinephrine for respiratory distress. The author highlights the importance of considering COVID-19 (severe acute respiratory syndrome coronavirus 2 (SARS-CoV-2)) as a potential etiology of croup in children. This distinction is crucial as such cases may necessitate more intensive medical intervention and prolonged monitoring compared to standard croup treatment protocols. The patient reported here did not require intensive care admission or respiratory support.

## Introduction

Laryngotracheitis (croup) in a child or an infant presenting with stridor is a common reason for an emergency department visit in the United States especially during fall and winter [1]. Herein, we report an uncommon case of croup secondary to coronavirus disease 2019 (COVID-19) infection in a three-year-old boy who presented with stridor and respiratory distress, necessitating hospitalization and three doses of racemic epinephrine. We would like to underline the significance of considering severe acute respiratory syndrome coronavirus 2 (SARS-CoV-2) as one of the causes of croup in children, as these patients will probably need respiratory support and longer monitoring than usual [1].

## Case presentation

A Hispanic three-year-old boy child with a known medical history of sickle cell trait and autistic spectrum disorder presented to the emergency department (ED) with a complaint of subjective fever for which he received oral acetaminophen and cough for two days. His sister had similar symptoms one day earlier, but she was not seen by a healthcare professional. The patient developed worsening shortness of breath four hours before presentation. There was no history of choking or foreign body inhalation, and the mother was not sure about exposure to someone with COVID-19 infection as her other kid was attending school. The patient attended a family gathering two days before the presentation. Mom denied skin rash, nausea, vomiting, diarrhea, constipation, abdominal pain, urinary changes, or change of level of consciousness, and he could maintain good hydration status. Upon evaluation, the child had audible biphasic stridor and suprasternal retractions, his temperature was 38.4 degrees Celsius, his heart rate was 120 beats per minute, his respiratory rate was 20 cycles per minute, and his oxygen saturation was 95% on room air; chest exam showed bilateral equal air entry. In the ED, he received 0.15 milligrams per kilogram of dexamethasone and one dose of 0.5 mL of 2.25% racemic epinephrine with mild improvement of the suprasternal retractions and persistent croup. He continues to have inspiratory and expiratory stridor, for which he received the second dose of racemic epinephrine one hour after the first one. The child's stridor improved. However, he continued to have stridor during exertion (crying). He was admitted to the pediatrics inpatient services for observation. A multiplex polymerase chain reaction (PCR) panel was negative, a nasopharyngeal swab tested positive for SARS-CoV-2 and negative for flu, and no blood work or imaging studies were done.

The infant maintained good oral intake during hospitalization; we closely monitored his vital signs. He developed a gradually worsening (first expiratory and then biphasic) stridor again within 10-12 hours after admission, for which he received another dose of racemic epinephrine. After the third dose of racemic epinephrine, his stridulous breathing almost disappeared, except for mild hoarseness during crying. We continued to monitor his breathing effort and his vital signs for 16 more hours after the third dose of epinephrine, then he was discharged home on no medications, but the parents were instructed to give him one oral dose of hydrocortisone should he develop any signs of shortness of breath or noisy breathing and bring him to the ED. A telehealth visit was scheduled the next day; the child was doing great and had no stridor during rest and exertion (playing).

## Discussion

Up to 4.1% of pediatric patients with COVID-19 in the United States required hospital admission [1], one-third (approximately 33%) required intensive care, and 6% needed invasive ventilation [2].

SARS-CoV-2 (COVID-19) infection in pediatrics has diverse presentations and variable severity ranging from asymptomatic illness to the novel multisystem inflammatory syndrome in children (MIS-C), which itself has a spectrum of presentation [3-5]. The most common presentations reported were upper respiratory tract infections with cough and fever [6]. Other presentations include pneumonia, respiratory failure that may require mechanical ventilation, and Kawasaki-like illness [3,4]. Most of the patients who required pediatric intensive care unit admission had underlying comorbid conditions, including underlying lung disease, congenital heart disease, or genetic syndromes [6]. Our patient presented with biphasic stridor and fever, and he had sickle cell trait and autism. While our patient lacked confirmatory bloodwork and may have had a co-infection, previous reports have documented more severe croup presentations in children with SARS-CoV-2 infection. Given this association, a link between our patient's clinical picture (biphasic stridor, fever) and potential COVID-19 infection is reasonable and cannot be entirely ruled out [7].

Croup as a clinical presentation of coronavirus infection is not new, as other coronaviruses have presented similarly [8]. On the other hand, croup as a COVID-19 presentation in pediatric patients was only reported in three articles (a total number of five patients) [9-11]. All the reported cases required hospital admission for close stridor monitoring and nebulization therapy besides dexamethasone, and one patient required non-invasive ventilation and heliox [9-11].

## Conclusions

Our case report adds to the body of evidence that croup is a presentation of pediatric COVID-19 infection and might be seen more often in the future. It is recommended that providers screen for COVID-19 infection in a patient presenting with clinical features consistent with croup. Patient care plans should include caregiver counseling on isolation precautions and monitoring for potential MIS-C development. Further studies are needed in the future, and there is a lot to learn about the relationship between COVID-19 infection and laryngotracheitis.
